# Athletæ

**Published:** 1847-10

**Authors:** 


"*Athletæ.*"—We copy the following from our spirited contemporary, the New-York Annalist, to whose extensive and well selected *varia* we are often much indebted for medical items. There is certainly no class of the community who, as a class, enjoy so little recreation as medical men; and there are none to whom recreation is more necessary. We are satisfied that both physicians and patients would do better, if more self-indulgence in this respect were practiced. The mind taxed by a constant succession of monotonous duties looses its energy, and becomes less capable of the active exercise of its faculties. The spirits and physical powers suffer. Physicians have occasions enough to appreciate the truth conveyed in the oft quoted phrase *mens sana in corpore sano*, but they are not less, perhaps even more neglectful of practical conformity to this maxim than others. In reply, then, to the question with which our esteemed *comfrere* closes his paragraph, we declare our readiness to advocate any thing which promises increased health, strength and happiness to those to whose interests our humble efforts are devoted. We *go in* also for any plan which will bring together members of our profession in social intercourse, and lead them to feel that common interests, pursuits, cares and enjoyments, should unite them in bonds of a true and loyal brotherhood.

"The late Dr. John Ramsbotham was a member of a club of twelve, calling themselves the "Athletæ," comprising Sir A. Cooper, Drs. Cooke, Lettsom, Babington, and Prof. Coleman. He was the last survivor; Sir A. Cooper the last but two. It was originally designed to give its members a little periodical relaxation out of town, to afford them all the means of healthy exercise, and some of them an opportunity of athletic display, in various games of strength and agility. At its commencement it was held at a tavern a short distance from London; afterward, as the members rose in public estimation, it was thought undignified to assemble at a common bowling green, and first Dr. Lettsom's elegant villa at Camberwell, then Dr. Babington,s country house at West Green, became the scene of their gatherings. Later still, when time had stiffened the joints of its founders, and rendered their sinews rigid, it deviated into a dining and card party every alternate week. It must have been a pleasing sight to see these eminent men laying aside for a time, the cares of science and the responsibilities of a professional life, and, in perfect good-fellowship, hats and coats off, and faces flushed with exertion, leaping, whooping, and struggling for the possession of a ball, vying in the race, or tumbling one another upon the greensward in the wrestle. Such a coterie might, we should think, find favor among us, and both presently and prospectively be a pleasing subject of conversation. Who goes in for it?"

